# Supplementary material for: Size-segregated analysis of PAHs in Urban air: Source apportionment and health risk assessment in an Urban canal-adjacent environment
Source: PLoS One. 2025 Apr 24;20(4):e0320405. doi: 10.1371/journal.pone.0320405 (PMC12021163; doi:10.1371/journal.pone.0320405)
Supplement: S1 Table — (DOCX) [file pone.0320405.s001.docx]

Table S1. The jet orifice dimensions and particle size ranges for each stage of TE-Cascade Impactor Series 10-8XX Viable Particle Sizing Instruments.

| Stage | Orifice Diameter (mm) | Range of Particle Sizes (Microns) |
| --- | --- | --- |
| 1 | 1.18 | 7.0 and above |
| 2 | 0.91 | 4.7-7.0 |
| 3 | 0.71 | 3.3-4.7 |
| 4 | 0.53 | 2.1-3.3 |
| 5 | 0.34 | 1.1-2.1 |
| 6 | 0.25 | 0.65-1.1 |

(<https://tisch-env.com/wp-content/uploads/2015/06/TE-10-800-Viable-Cascade-Impactor.pdf>)
