# Supplementary material for: Size-segregated analysis of PAHs in Urban air: Source apportionment and health risk assessment in an Urban canal-adjacent environment
Source: PLoS One. 2025 Apr 24;20(4):e0320405. doi: 10.1371/journal.pone.0320405 (PMC12021163; doi:10.1371/journal.pone.0320405)
Supplement: S2 Table — (DOCX) [file pone.0320405.s002.docx]

Table S2. Input data statistics for PMF analysis.

| Species | Category | S/N | Min | 25^th^ | Median | 75^th^ | Max | % Modeled Samples | %  Raw Samples |
| --- | --- | --- | --- | --- | --- | --- | --- | --- | --- |
| Phe | Strong | 0.067965118 | 0 | 0 | 0 | 25.5 | 943 | 100.00% | 100.00% |
| An | Strong | 0.981074503 | 0 | 0 | 54.5 | 230.5 | 2331 | 100.00% | 100.00% |
| Fluo | Strong | 0.57183656 | 0 | 49 | 168.5 | 367 | 2170 | 100.00% | 100.00% |
| Pyr | Strong | 1.205469072 | 0 | 64 | 207 | 394.75 | 2184 | 100.00% | 100.00% |
| B[a]A | Strong | 0.527058976 | 0 | 0 | 16 | 143.5 | 1654 | 100.00% | 100.00% |
| Chry | Strong | 0.417773973 | 0 | 0 | 21.5 | 175.25 | 1698 | 100.00% | 100.00% |
| B[b]F | Strong | 3.270272519 | 0 | 23.75 | 311.5 | 901.25 | 4387 | 100.00% | 100.00% |
| B[k]F | Strong | 0.892441173 | 0 | 37 | 147 | 602 | 3279 | 100.00% | 100.00% |
| B[a]P | Strong | 0.492544368 | 0 | 0 | 0 | 123.75 | 1655 | 100.00% | 100.00% |
| Ind | Strong | 0.080079648 | 0 | 0 | 0 | 0 | 722 | 100.00% | 100.00% |
| D[a,h]A | Strong | 0.036006547 | 0 | 0 | 0 | 0 | 537 | 100.00% | 100.00% |
| B[g,h,i]P | Strong | 0.256237639 | 0 | 0 | 0 | 46.75 | 897 | 100.00% | 100.00% |
