# Supplementary material for: Size-segregated analysis of PAHs in Urban air: Source apportionment and health risk assessment in an Urban canal-adjacent environment
Source: PLoS One. 2025 Apr 24;20(4):e0320405. doi: 10.1371/journal.pone.0320405 (PMC12021163; doi:10.1371/journal.pone.0320405)
Supplement: S3 Table — (DOCX) [file pone.0320405.s003.docx]

Table S3. Base run summary table of PMF analysis.

| Run # | Q_(Robust)_ | Q_(True)_ | Converged | # Steps | Q_(True)/_Q_(Exp)_ |
| --- | --- | --- | --- | --- | --- |
| 1 | 648 | 666 | Yes | 289 | 1.000705719 |
| 2 | 648 | 666 | Yes | 211 | 1.000705719 |
| 3 | 648 | 666 | Yes | 183 | 1.00070715 |
| 4 | 648 | 666 | Yes | 225 | 1.000705719 |
| 5 | 648 | 666 | Yes | 288 | 1.000704169 |
| 6 | 648 | 666 | Yes | 284 | 1.000704169 |
| 7 | 713 | 749 | Yes | 323 | 1.124900937 |
| 8 | 648 | 666 | Yes | 248 | 1.000708699 |
| 9 | 648 | 666 | Yes | 338 | 1.000704169 |
| 10 | 648 | 666 | Yes | 186 | 1.000704169 |
| 11 | 648 | 666 | Yes | 227 | 1.000696659 |
| 12 | 648 | 666 | Yes | 162 | 1.000711679 |
| 13 | 648 | 666 | Yes | 307 | 1.000702739 |
| 14 | 648 | 666 | Yes | 349 | 1.000704169 |
| 15 | 648 | 666 | Yes | 321 | 1.000702739 |
| 16 | 648 | 666 | Yes | 217 | 1.000708699 |
| 17 | 648 | 666 | Yes | 335 | 1.000708699 |
| 18 | 648 | 666 | Yes | 220 | 1.000702739 |
| 19 | 648 | 666 | Yes | 134 | 1.000716209 |
| 20 | 713 | 749 | Yes | 224 | 1.124896407 |
