# Supplementary material for: Size-segregated analysis of PAHs in Urban air: Source apportionment and health risk assessment in an Urban canal-adjacent environment
Source: PLoS One. 2025 Apr 24;20(4):e0320405. doi: 10.1371/journal.pone.0320405 (PMC12021163; doi:10.1371/journal.pone.0320405)
Supplement: S1 Appendix — (DOCX) [file pone.0320405.s004.docx]

Appendix I.

**Cascade impactor principle**

The instrument used for sample collection was a TE-10-800 cascade impactor. Cascade impactors are specialised air-sampling instruments that use size-based separation to capture and sort airborne particles. Cascade impactors use a series of nozzles and impaction surfaces to cause particles of varying sizes to deposit or impact the collection substrates at various stages (see Table S1). The obtained size-resolved particle samples are crucial for a variety of applications, such as air quality monitoring, environmental research, and aerosol characterisation.

Below is a simple explanation of a cascade impactor's sampling principle:

Particle Entry: Particles of various sizes are attracted to the cascade impactor via air. To determine airflow, a vacuum pump or air sampling system is typically employed.

Nozzles and Stages: The cascade impactor comprises several stages, each of which is outfitted with a nozzle that accelerates the incoming air to a specific velocity. The nozzles are designed to preserve the laminar flow conditions while maintaining a consistent flow rate.

Impaction and Separation: As air moves through each step, it comes into contact with the impaction surface, which is usually a flat or curved substrate. Because of their inertia, the particles in the air stream continue in a straight line, whereas the air changes direction around the impaction surface. This causes particles of various sizes to collide with the surface. Larger and heavier particles are more likely to collide, whereas tiny particles continue to move through the air.

Particle Collection: Each impaction surface is designed to capture particles of various sizes. Thus, the impactor separates the particles by size, with the largest particles deposited in the first stage and increasingly smaller particles deposited in successive stages. Following sampling, the collected substrates from each stage are removed and can be examined to determine the particle size distribution. Depending on the research aim, several analytical procedures, such as microscopy, chemical analysis, and gravimetry, can be used for this.

Cascade impactors offer size-resolved data on particle concentrations and can be applied for studying particle distributions in the atmosphere or specialised situations. Researchers have utilised size-resolved data to estimate the health risks associated with different particle sizes, identify pollution sources, and assess the success of air quality control strategies. Cascade impactors are available in a variety of configurations, including multistage and single-stage impactors, each suitable for a specific use. The impactor design and stage selection were determined based on the research objectives and particle size range of interest.

**Materials and Tools:**

The materials required were as follows:

- Impactor with a 6-stage cascade
- Air sampling pump with impactor-calibrated flow rate
- Particle collecting substrates (i.e., quartz fibre filters)
- Substrate sample holders or cassettes
- Tubing and fittings
- Personal protection equipment (PPE) (e.g. gloves, lab coat, safety goggles), as needed
- Calibration device (for calibrating flow rates)

**Procedure:**

The cascade impactor and all accompanying equipment were cleaned and confirmed to be functioning. The manufacturer's instructions were followed to calibrate the air sampling pump to the specified flow rate required by the cascade impactor. Suitable substrates (i.e., a quartz fibre filter) were prepared by cutting them to suit the impactor stages or using pre-cut substrates.

**Setup:**

The air-sampling pump was connected to the inlet of the cascade impactor using appropriate tubing and connectors. The substrate was placed in each stage of the impactor. All seals and connections were airtight to maintain a consistent and uniform flow rate.
