# Supplementary material for: Size-segregated analysis of PAHs in Urban air: Source apportionment and health risk assessment in an Urban canal-adjacent environment
Source: PLoS One. 2025 Apr 24;20(4):e0320405. doi: 10.1371/journal.pone.0320405 (PMC12021163; doi:10.1371/journal.pone.0320405)
Supplement: Attachment — Submitted filename: Comments.docx [file pone.0320405.s005.docx]

**Comments:**

Dear editor of “***PLOS ONE***”

Thank you for inviting me to review the manuscript titled “**The Influence of Canal Boat Emissions on the Particle Size Distribution of PAHs in the Ambient Air of Bangkok**”. The manuscript was carefully studied. There are minor comments which present below.

1. The title of the article does not reflect the purpose of the manuscript well, it should be corrected
2. In the abstract, the purpose of the article is not well stated.
3. Keywords are not chosen correctly and need to be revised.
4. The manuscript is grammatically flawed.
5. The map of the sampling location and the boat channel crossing should also be specified.
6. What is the distance between Air Quality Observatory Site (NAQOS) and the boat channel crossing?
7. The order of the sampling method, especially the headings selected in this section, is disproportionate and needs to be corrected.
8. The duration of sampling, weather conditions (wind, precipitation and temperature) have not been stated
9. PAHs are ubiquitous compounds, so they may be present even in clean air. Considering the height and distance of the sampler from their passage, how can these PAHs be related to the boat channel?
10. The volume of results and discussion is large, it is better to write more briefly.
11. There are a lot of tables, the ones that don't need to be deleted or replaced with diagrams
