# Supplementary material for: Size-segregated analysis of PAHs in Urban air: Source apportionment and health risk assessment in an Urban canal-adjacent environment
Source: PLoS One. 2025 Apr 24;20(4):e0320405. doi: 10.1371/journal.pone.0320405 (PMC12021163; doi:10.1371/journal.pone.0320405)
Supplement: Attachment — Submitted filename: Response to reviewer-170125.docx [file pone.0320405.s008.docx]

6. Review Comments to the Author

Please use the space provided to explain your answers to the questions above. You may also include additional comments for the author, including concerns about dual publication, research ethics, or publication ethics. (Please upload your review as an attachment if it exceeds 20,000 characters)

Reviewer #2: The authors have adequately addressed my comments raised in a previous round of review and feel that this manuscript is now acceptable. The manuscript technically sound with appropriate statistical analysis. The authors made all data underlying the findings in their manuscript fully available. The manuscript presented in an intelligible fashion and written in standard English.

Response to reviewer’s comment

Thank you very much for your positive feedback and for recognizing the improvements made in the manuscript. We sincerely appreciate your thorough review during the earlier stages and are grateful for your valuable comments, which significantly enhanced the quality and clarity of our work. We are pleased to know that you find the manuscript technically sound, with appropriate statistical analysis, and presented in an intelligible fashion. Your acknowledgment of the availability of underlying data and the standard of English used in the manuscript is also greatly appreciated. Thank you once again for your constructive input and kind words throughout the review process.

Please see the attachment for additional suggestions.

Reviewer #4: The authors addressed most of the comments from the reviewers. I have a few remarks on PMF source apportionment results and data summary.
Lines 665-706. The source profiles from PMF modeling are clear. However, the authors did not present the contribution of individual sources to total PAHs. One reference is recommended.
<https://doi.org/10.3390/atmos15030346>
The authors could read the reference and organize the data as those in the recommended reference.

Response to reviewer’s comment

Line-755-782: The presentation of the contribution of individual sources to total PAHs was clear discussed. The recommended study was also cited please kindly check.

Another issue is Figure 2. I recommend the authors plot a figure of size distributions of LMW PAHs (a), MMW PAHs (b), and HMW PAHs (c), as shown in the recommended reference.

Response to reviewer’s comment

Already revised as shown in Line-541-571 and Fig.2. Please kindly check.

Reviewer #5: Here are pointwise comments for improving the given article:

Response to reviewer’s comment

We sincerely appreciate the insightful and constructive comments provided by the reviewers, which have significantly contributed to improving the quality and clarity of our manuscript. Their valuable feedback has helped us refine our analysis, strengthen our arguments, and ensure the robustness of our findings.

1. Provide a clearer introduction that outlines the significance of studying PAH concentrations in urban environments. This will help contextualize the research for readers unfamiliar with the topic.

Response to reviewer’s comment

Already revised as suggest. Please check Line-60-108.

2. Methodology
- Expand on the methodology used for sampling and analysis of PAHs. Include specifics on the sampling duration, frequency, and any analytical techniques employed (e.g., mass spectrometry) to enhance reproducibility.

Response to reviewer’s comment

The specifics on the sampling duration and frequency were mentioned in Line-125-128 “A total of 66 air samples (i.e., 6×11) were collected continuously from 9:00 AM Monday to 9:00 AM Saturday over an 11-week period, amounting to a total sampling duration of 120 hours per week, to ensure consistency and capture potential variations in particulate composition across weekdays.” Analytical techniques were clearly written in the Line-224-240 and Line-255-278.

3.Elaborate on the statistical methods applied to analyze the data, including any software used, and how the results were interpreted. This will strengthen the credibility of the findings.

Response to reviewer’s comment

**Statistical Methods and Data Interpretation**

**Preprocessing and Normalization**

The dataset was preprocessed to ensure accuracy and consistency prior to statistical analysis. Outliers were removed to minimize noise and ensure the reliability of the dataset. Concentrations of PAH congeners across six particle size fractions were normalized to prevent any variable from disproportionately influencing the results, ensuring that all variables contributed equally to the analysis.

**Principal Component Analysis (PCA)**

Principal Component Analysis (PCA) was utilized to reduce the dimensionality of the dataset and identify significant patterns in the data. PCA highlighted key variables that accounted for most of the variance in the dataset. Principal components (PCs) with eigenvalues greater than 1 were retained, and varimax rotation was applied to enhance the interpretability of the results. Each PC represented a distinct combination of variables linked to potential PAH sources. For instance, PC1, characterized by high loadings of fluoranthene, pyrene, and benzo[g,h,i]perylene, was associated with gasoline emissions. These PCs provided insights into the dominant sources of PAHs in the study area, including traffic emissions, industrial activities, and biomass burning.

**Positive Matrix Factorization (PMF)**

The PMF model, implemented using the "EPA PMF 5.0" software, was applied to apportion sources of PAHs. This method decomposed the input data matrix into source profiles and contribution matrices, incorporating uncertainty estimates to address measurement errors. Multiple runs (n=100) were conducted to ensure the stability and robustness of the results, and the model was optimized by testing 3–8 factors to identify the best solution. Bootstrap uncertainty prediction confirmed the reliability of the model, with mapping rates exceeding 90%. PMF revealed three major PAH sources: light vehicle emissions (34.2%), biomass combustion (20.4%), and diesel emissions (45.4%), with diesel emissions contributing significantly to finer particle fractions.

**Pearson Correlation Coefficients (PCCs)**

Pearson Correlation Coefficients (PCCs) were calculated to explore relationships between individual PAHs, aiding in identifying shared emission sources. Strong positive correlations, such as those observed between fluoranthene and pyrene (r=0.982), indicated common sources like vehicular emissions. Moderate correlations further supported the influence of mixed sources, including traffic and industrial activities.

**Hierarchical Cluster Analysis (HCA)**

Hierarchical Cluster Analysis (HCA) was employed to group PAH congeners based on their concentration profiles. Using agglomerative clustering and Euclidean distance, the dendrogram revealed two primary clusters. Cluster I included benzo[b]fluoranthene, while Cluster II contained other PAHs, such as phenanthrene and benzo[a]pyrene, reflecting their shared origins, such as traffic and combustion sources. Subclusters highlighted subtle variations in source contributions.

**Health Risk Assessment**

The Lifetime Lung Cancer Risk (LLCR) was computed to evaluate health risks associated with PAH exposure across different particle sizes. Toxic equivalency (TEQ) values were calculated using established toxic equivalence factors (TEFs) for each PAH, emphasizing the dominance of finer particles (e.g., PM2.1–3.3) in posing health risks due to their higher PAH concentrations. The LLCR values were well below the benchmark level of 1×10⁻⁶, indicating minimal cancer risks in the study area.

**Software and Tools**

All statistical analyses were conducted using well-established software. PCA and HCA were implemented using SPSS (IBM SPSS Statistics, Version 26), while PMF was executed with EPA PMF 5.0 software, following the recommendations in the EPA PMF User Guide. Data visualization, including dendrograms and source contribution plots, was performed using OriginPro and Microsoft Excel.

**Interpretation of Results**

The combined application of PCA and PMF provided a comprehensive understanding of PAH sources, while PCCs and HCA reinforced the source apportionment findings. The statistical methods highlighted the predominance of diesel emissions in finer particles, underscoring their health implications and the necessity for targeted emission control policies. These results establish a robust framework for air quality management and health risk mitigation in urban environments.

4. Add comparisons of the findings with similar studies in other urban areas to contextualize the PAH concentrations reported in Bangkok, which can highlight the uniqueness or commonality of the findings.

Response to reviewer’s comment

Already revised as suggest. Please kindly check in Line-499-532.

5. Discuss the methodologies utilized for identifying the sources of PAHs, such as receptor modeling or statistical source apportionment. Detailed source attribution can enhance understanding of pollution dynamics.

Response to reviewer’s comment

The study utilized advanced methodologies, including receptor modeling and statistical source apportionment techniques, to identify and quantify the sources of PAHs across six particle size fractions. These approaches provided a detailed understanding of pollution dynamics and the relative contributions of various sources, such as vehicular emissions, industrial activities, and biomass combustion.

**Principal Component Analysis (PCA)**

PCA was employed as an initial step to reduce the dimensionality of the dataset and identify underlying patterns. By analyzing the chemical composition of PAHs in the particulate fractions, PCA grouped variables with similar behavior and attributed them to specific emission sources. Principal components (PCs) with eigenvalues greater than 1 were retained, and varimax rotation was applied to improve interpretability. For instance, PC1, dominated by fluoranthene, pyrene, and benzo[g,h,i]perylene, was linked to gasoline combustion, while PC2, characterized by benzo[a]anthracene and chrysene, indicated contributions from gasohol emissions. This analysis highlighted the dominance of traffic emissions as a primary source, with other PCs pointing to industrial processes and biomass burning.

**Positive Matrix Factorization (PMF)**

PMF, a receptor model, was used to complement PCA by providing quantitative source apportionment. The "EPA PMF 5.0" software was applied to the dataset, incorporating uncertainty estimates to account for measurement variability. The PMF model decomposed the data into source profiles and contributions, identifying three key sources: diesel emissions (45.4%), light vehicle exhaust (34.2%), and biomass combustion (20.4%). PMF was particularly effective in resolving overlapping sources by integrating uncertainty and ensuring non-negativity in source profiles, thus producing reliable and interpretable results. The model’s findings were validated using bootstrap uncertainty prediction, which confirmed the stability of the source contributions.

**Diagnostic Ratios and Hierarchical Cluster Analysis (HCA)**

Diagnostic binary ratios of PAHs, such as fluoranthene/pyrene (Fl/Pyr) and indeno[1,2,3-cd]pyrene/benzo[g,h,i]perylene (Ind/B[g,h,i]P), were calculated to infer potential emission sources. These ratios provided qualitative insights into whether PAHs originated from petroleum combustion, biomass burning, or mixed sources. Additionally, HCA was employed to group PAHs with similar concentration patterns into clusters, visualized through dendrograms. This method further reinforced the identification of shared sources, with clusters aligning well with combustion-related PAHs, such as those from traffic and industrial activities.

Collectively, these methodologies offered a comprehensive and robust framework for source attribution, enabling the study to disentangle complex pollution dynamics. The integration of PCA, PMF, diagnostic ratios, and HCA enhanced the understanding of how different sources contribute to PAH concentrations, supporting the development of targeted mitigation strategies to improve urban air quality.

6. Provide further explanation of the LLCR values, including how these were calculated and their implications. Consider including a brief discussion on the thresholds that determine health risks.

Response to reviewer’s comment

Already revised as suggest. Please kindly check in Line-837-850.

7. Engage in a more in-depth discussion on the implications of the findings. Discuss potential public health policies or interventions that could mitigate PAH exposure based on the identified sources.

Response to reviewer’s comment

Already revised as suggest. See Line-864-883.

8. Strengthen the conclusion by summarizing key findings and suggesting practical recommendations for policymakers or urban planners based on the study results.

Response to reviewer’s comment

Already revised as suggest. Please kindly check in Line-886-920.

9. Acknowledge any limitations in the study's design or scope, such as potential unmeasured sources of PAHs or limitations in spatial coverage.

Response to reviewer’s comment

Already revised as suggest. Please kindly check in Line-896-905.

10. References: There are fewer numbers of new references or recent references especially related to urban PAHS /health risk similar studies. Therefore, for current study should be given a strong impact if you can cite the following reference.
- The health risk and source assessment of polycyclic aromatic hydrocarbons (PAHs) in the soil of industrial cities in India
- Distribution, risk assessment, and source apportionment of polycyclic aromatic hydrocarbons (PAHs) using positive matrix factorization (PMF) in urban soils of East India
- Temporal variability of atmospheric particulate-bound polycyclic aromatic hydrocarbons (PAHs) over central east India: sources and carcinogenic risk assessment

Response to reviewer’s comment

Already cited as recommended in Line-76-77.
